# The effect of dot size in random-dot stereograms on the results of stereoacuity measurements

**DOI:** 10.1186/s12886-020-01526-6

**Published:** 2020-06-24

**Authors:** Lingzhi Zhao, Huang Wu

**Affiliations:** 1 Department of Medical Equipment, the Second Hospital of Jilin University, Changchun, China; 2 Department of Optometry, The Second Hospital of Jilin University, No. 218, Ziqiang Street, Nanguan District, Changchun, 130041 China

**Keywords:** Stereopsis, Random-dot stereograms, Smartphone

## Abstract

**Background:**

This study aimed to evaluate the effect of the size of the dots in random-dot stereograms on the results of stereoacuity measurements.

**Methods:**

A stereopsis measurement system was created using a phoropter and two 4 K smartphones. Three dot sizes, including 1 × 1 pixel, 6 × 6 pixels, and 10 × 10 pixels (equivalent to 0.17 min arc, 1 min arc, and 1.68 min arc, respectively), were used to form random-dot arrays, and each test pattern had one Lea symbol hidden within it. The resulting stereograms were tested on 30 subjects with normal acuity and stereoacuity.

**Results:**

Stereoacuity measured with the 1-pixel dots was significantly worse than that measured with the 6-pixel dots (Wilcoxon signed-rank test, Z = -4.903, *P* < 0.001) and the 10-pixel dots (*Z* = -4.941, *P* < 0.001). No significant difference was found between 6-pixel dot and 10-pixel dot stereograms (*Z* = -1.000, *P* = 0.317).

**Conclusion:**

The size of the dots in random-dot stereograms affects the test results significantly when the dots are too small for the eye to resolve.

## Background

Random-dot stereograms (RDS) are widely used in the clinical evaluation of stereopsis. The advantage of this technique is that it eliminates monocular clues more thoroughly than contour based stereo tests. In clinical practice, the stereopsis to detect contour-based stereo target, e.g. the circle pattern in the Fly Stereo Acuity Test (Vision Assessment Corporation, Illinois, USA), is considered as ‘local’, while the stereopsis to detect random dot-based stereo target, e.g. the Pacman hidden in TNO stereotest (Lameris Ootech BV, Ede, Netherlands), is considered as ‘global’ [1]. The neural processing of ‘local’ and ‘global’ stereopsis may be different. However, the two mechanisms cannot be entirely separated [2]. In neurophysiology research, local and global stereograms may be determined by the number of dots contained within the stereogram. For example, ‘Local’ stereopsis is traditionally defined by the use of a small number of dots to form a pattern, while ‘global’ stereopsis involves the use of many dots to form a pattern. From this point of view, a test pattern consists of a small number of random dots and may also be considered ‘local’ [3]. In Gantz’s research, the density of RDSs lower than 0.39% was considered a ‘local’ stereo target; otherwise, it was considered a ‘global’ stereo target [4].

In the Frisby stereotest (Stereotest Ltd., Sheffield, UK) [1, 5], the subject is asked to identify a shape hidden in an array of randomly arranged triangles of varying sizes. The shape is distinguished by a real difference in depth and can be perceived without the help of any other appliance. In the random-dot E stereo test (Vision Assessment Corporation, Illinois, USA) [1, 5], the letter E is hidden in a random dot array, but can be perceived with the aid of polarising spectacles that divide the images seen by the two eyes. In the TNO test [1, 5], the test shapes are disks with a missing sector and the two stereo elements printed in red and green for viewing with red/green anaglyph stereo glasses. Although the measurements are all based on detecting the minimum disparities a subject is able to distinguish, the test result evaluated with different stereotests may differ from each other [6]: For example, the stereoacuity measured with the TNO test is worse than the acuity measured with other methods, either in a normal population [7] or in patients with abnormal binocular vision [8]. The mechanisms underlying these differences have not been clearly established [9].

Several researchers have discussed whether dot size in a RDS could affect the test result. In Henriksen’s study, the size of half-matched RDSs (half the dots in RDSs are correlated and half are anticorrelated) was set at 0.025°, 0.05° and 0.075° respectively. They found that psychophysical performance decreases with smaller dot size, and stated that smaller dots might decrease the local correlation variability [10]. A previous study has confirmed that increasing dot size may improve accuracy in detecting binocular disparity - slightly, but significantly [11].

Whatever the test symbol or the test procedure, the RDSs used in the clinic are simpler than those conducted in a laboratory environment. We adopted our newly designed stereoacuity measurement system, a phoropter combined with two 4 K smartphones [12], to explore the effect of dot size in RDS. Extremely small dots, far smaller than the recognition resolution of the human eye, were utilized. For some stereotests, e.g. Random dot E test, the examined distance could be prolonged to obtain a finer stereo threshold. The visual angle of the dots in the background may reduce to an unrecognizable level. The aim of this study was to evaluate the possible difference of stereopsis tested with RDSs composed of different dot sizes.

## Methods

### Evaluation of stereoacuity with RDSs of different dot size

#### Test system

**Fig. 1:**
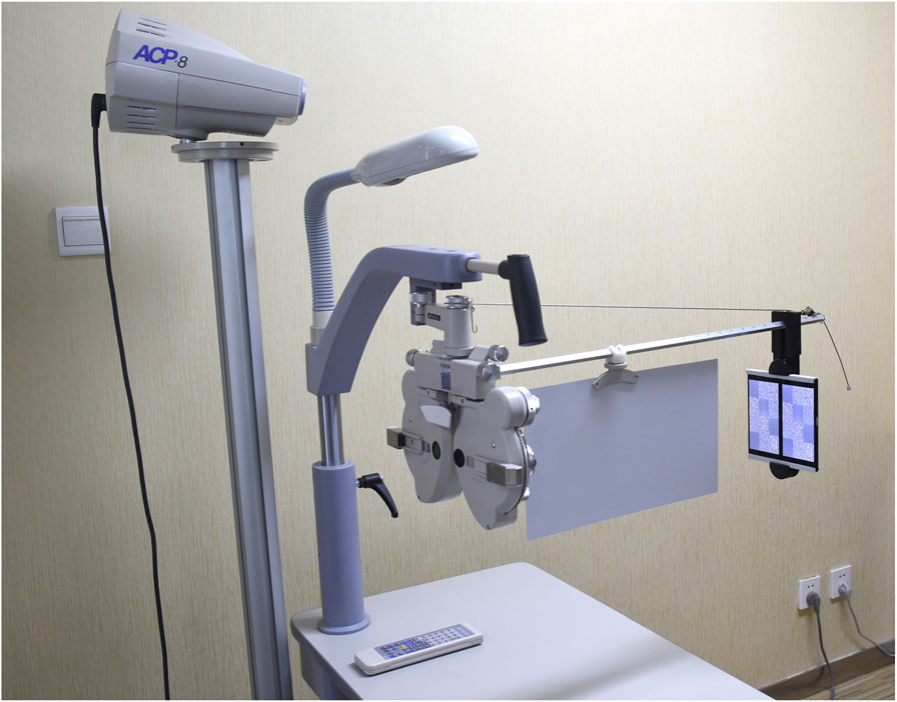
Stereoacuity measurement system. A test system consisted of a phoropter and two 4 K smartphones

A novel stereopsis measurement system was developed, using a phoropter (VT-10, Topcon Corp, Tokyo, Japan) and two Sony smartphones (Sony Xperia Z5 Premium Dual E6883, Sony Mobile Communications Inc. Tokyo, Japan) (Fig. 1). At a test distance of 65 cm with this system, a 1-px (pixel) disparity of two images equates to an angular disparity of 10″ (arcsec, second of arc), which makes it possible to test stereoacuity with a resolution of 10″.

#### Test symbols

A program written in C# was used to produce all test targets. The test symbol imitated the Random Dot 3 Stereo Acuity Test (Vision Assessment Corporation, Illinois, USA). Lea symbols (solid filled symbols of a house, circle, square and apple) were used as the test targets, hidden within random-dot stereograms. The random dots were chosen to be square rather than circular because of the limitations of the smartphones’ screen pixel arrangement. Black dots and white dots constituted test pictures, and the proportion of them was 1:1 (dot density 50%).

**Fig. 2:**
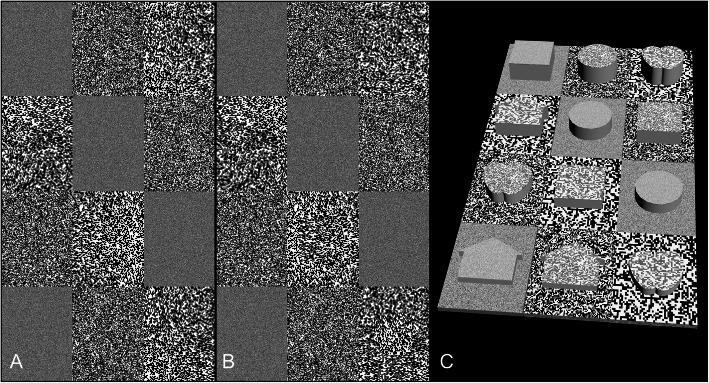
Legend of test image 1 (8 px to 5 px, equivalent to 80″ to 50″ at 65 cm). This image contains 12 rectangles (720 × 960 px). The disparities are set at 8 px in the first line (the top 3 rectangles); 7px in the second line; 6px in the third line; and 5px in the fourth line. The dot size is 1px, 6px and 10px in the first line; 10px, 1px and 6px in the second line; 6px, 10px and 1px in the third line; 1px, 6px and 10px in the fourth line. The size of the test symbol is 460 × 460 px for the circle, square and apple; the main body of the house is also 460 × 460 px with the eaves extending another 50px on the left and right side, respectively. The target symbols hidden in the rectangles are the square, circle and apple in the first line; the house, circle and square in the second line; the apple, square and circle in the third line; the house, house and apple in the fourth line. **a** is seen by the left eye and **b** is seen by the right eye. **c** is the simulation of the percepts generated by the test images **a** and **b**. This is an attempt to simulate what a subject might perceive when fusing **a** and **b** as one image. The stereo symbols appear to pop out of the background plane

Three dot sizes were used to construct the random-dot arrays, including 1-px (1 × 1 px, equivalent 0.17 min arc at 65 cm, similarly hereinafter), 6-px (6 × 6 px, 1 min arc), and 10-px (10 × 10 px, 1.68 min arc). Each test pattern included a symbol hidden within it. Two test images were created. The first image included four lines ranging from 8-px disparity to 5-px disparity, while the second image included four lines ranging from 4-px disparity to 1-px disparity. Image details are shown in Fig. 2.

#### Test procedure

The subjects were examined with an auto-refractor (KR-8900PA, Topcon Corp, Tokyo, Japan) and a phoropter to determine the optimal refractive correction and the best corrected visual acuity (BCVA), after which the two 4 K smartphones were attached to the near vision rod. The test distance was 65 cm. With the aid of two 5.5^Δ^ base out Risley prisms, two smartphones could be fused as one image (Fig. 1). The subjects were asked to identify the forms hidden in the random dot stereograms, from left to right and top to bottom. The last correct answer for each size of dot was recorded as the stereoacuity of the subject.

#### Subjects

Thirty subjects were enrolled, 11 men and 19 women, with ages of 20–31 (25.3 ± 3.8) years. The corrected visual acuity of each eye was at least 0 logMAR, and the stereoacuity was at least 40″, as evaluated by the Fly Stereo Acuity Test. The study was conducted at the Second Hospital of Jilin University in China. The research protocol observed the tenets of the Declaration of Helsinki and was approved by the ethics committee of the Second Hospital of Jilin University (No. 2017–89).

#### Statistical analysis

All data were processed using PASW Statistics 18.0 (IBM SPSS Inc.). Because the data did not satisfy tests for normality of distribution, nonparametric tests were carried out. The Friedman test was used to analyse the differences among the groups, with *P* < 0.05 used as the threshold for statistical significance. The Wilcoxon signed-rank test was used to compare the differences between pairs of groups. Because three comparisons were conducted, *P* < 0.017 (0.05/3) was used as the threshold for statistical significance [13].

### Measurement of the dot size of RDSs utilized in the clinic

The TNO stereotest, Randot Stereotest (Stereo Optical Company, Inc. Illinois, USA), Butterfly Stereo Acuity Test (Vision Assessment Corporation, Illinois, USA), Pass Test 3 (Vision Assessment Corporation, Illinois, USA), Random Dot E Stereotest, Random Dot Stereo Acuity Test (Vision Assessment Corporation, Illinois, USA), Random Dot 2 Stereo Acuity Test (Vision Assessment Corporation, Illinois, USA), and Random Dot 3 Stereo Acuity Test were chosen. A scanner (ScanMaker S260, Microtek International, INC. Shanghai, China) was used to scan the pictures in a resolution of 3200 × 3200. The TNO stereotest was scanned directly. The other 7 tests were all based on polarizing technique; this means that the tested image contains two polarization pictures and the polarization direction of each image is perpendicular to the other. The test card was covered by a polarizing glass plate and scanned twice. The polarizing orientation of the glass was perpendicular during the two scans, thereby keeping the same polarization direction as original images. Two clear images would be decomposed in this manner. A 600 × 600 frame was used to cut the image 10 times randomly in the random dots background region, and to find the smallest diameter of the dots in that area. The size of the dot was measured by counting the number of pixels occupied in the narrowest diameter.

The specifically images selected for analysis were as follows: TNO stereotest, 19th edition, the first image for plate V; Randot Stereotest, the first image in large homogeneous areas; Butterfly Stereo Acuity Test, the random dot pattern for the butterfly; Pass Test 3, the 60″ test card; Random Dot E Stereotest, the card with the letter E; Random Dot Stereo Acuity Test, the first image in section A, page 1; Random Dot 2 Stereo Acuity Test, the first image in section A, page 1; Random Dot 3 Stereo Acuity Test, the first circle in section A, page 2. The measurement method relied on subjective judgment because the forms of the dots were different from test to test. For instance, the dots in the background of the TNO stereotest were irregular. Therefore, the examiner could not find a regular circle or a square, but had to identify the smallest dot in the view window and measure the narrowest diameter.

## Results

### Stereoacuity evaluation of RDSs of different dot sizes

**Fig. 3:**
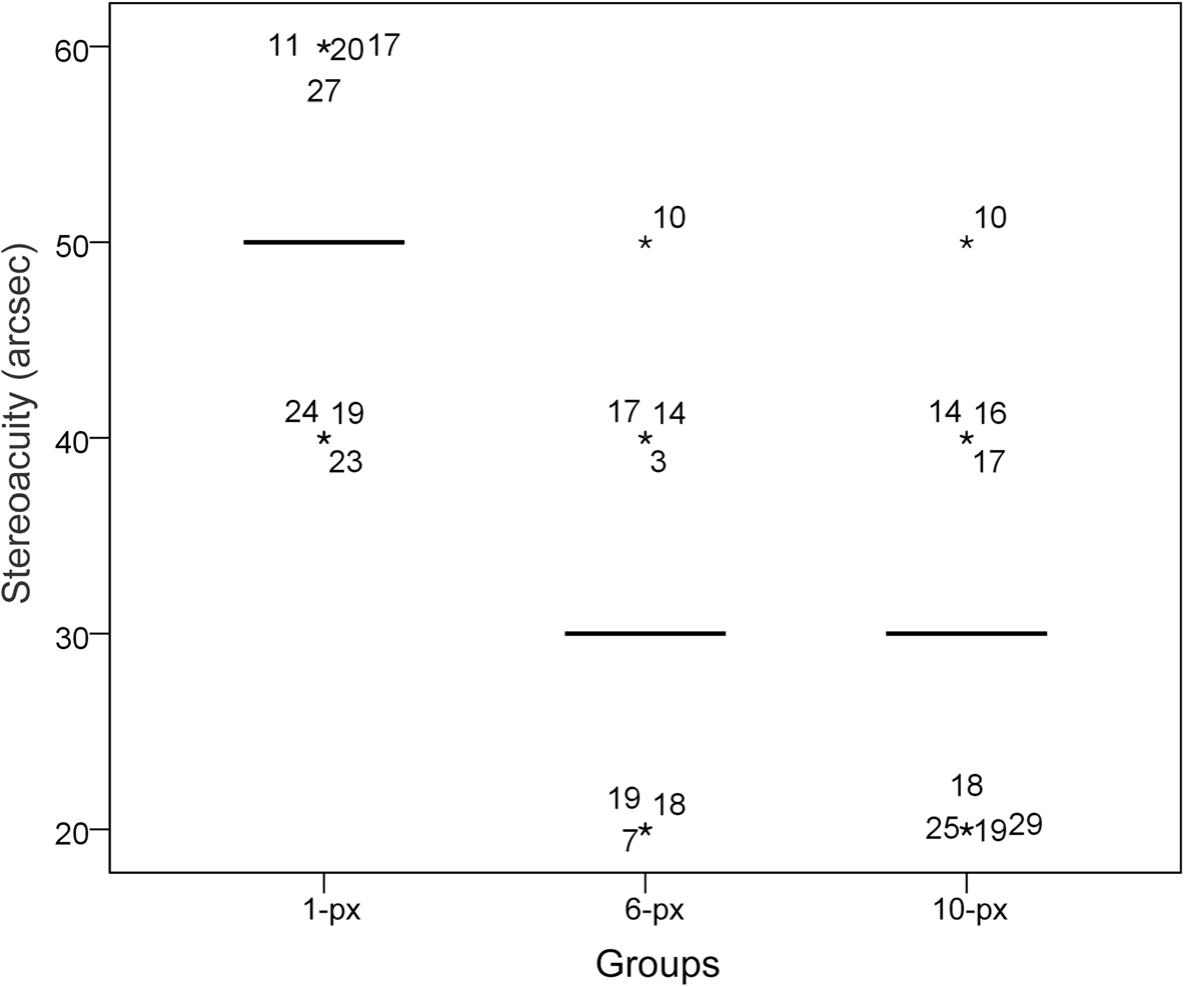
Boxplot of the stereoacuity of three groups. The interquartile range of the three groups was all equal to zero, so the body of the box was changed to a line (the first quartile, the median and the third quartile were the same). Asterisks represented the test value which was beyond the scope of the interquartile range. The numbers located beside the asterisks were the codes of the participants. The median and interquartile range (M [QR]) of 1-px, 6-px, and 10-px were 50 [0], 30 [0], and 30 [0], respectively

The median stereoacuities for the 1-px group, 6-px group, and 10-px group were 50″, 30″, and 30″, respectively (Fig. 3). A significant difference was found among the three groups (Friedman test, *Chi-square* = 57.532, *P* < 0.001). The Wilcoxon signed-rank test was used to detect differences between the groups: 1-px group versus 6-px group, *Z* = -4.903, *P* < 0.001; 1-px group versus 10-px group, *Z* = -4.941, *P* < 0.001; 6-px group versus 10-px group, *Z* = -1.000, *P* = 0.317. Therefore, the stereoacuity of the 1-px group was significantly worse than those of the other two groups, while no significant difference was found between the 6-px and 10-px groups.

### Measurement of the dot size of RDSs utilized in the clinic

The measured results are shown in Table 1. At a distance of 40 cm, the visual angle of the smallest diameter of a dot located in the background was from 0.8 min arc to 1.8 min arc.

**Table 1:** The dot size in 8 stereotests

Name of Stereotest	Dot Size	
	mm	min arc at 40 cm
TNO stereotest	0.20	1.8
Randot Stereotest	0.10	0.8
Butterfly Stereo Acuity Test	0.15	1.3
Pass Test 3	0.12	1.0
Random Dot E Stereotest	0.13	1.1
Random Dot Stereo Acuity Test	0.13	1.1
Random Dot 2 Stereo Acuity Test	0.13	1.1
Random Dot 3 Stereo Acuity Test	0.09	0.8

## Discussion

The random-dot stereogram is the most used method of evaluating stereoacuity. Several tests widely used in research or the clinic are based on this technique. Different designs of the test shapes, or differences in the random dots, may lead to differences in the results of each method. In Simon’s view, the poorer stereoacuity measured by the Frisby test may be caused by the widely spaced random elements that make the contour of the stereofigure discontiguous with the surrounding pattern [14]. Gantz and Bedell found that the effect of dot density on stereothresholds was significant [4]. However, dot size is another factor that may affect the test result [3, 11]. Forty years ago, Pitblado studied cerebral asymmetry with the aid of RDS. For the recognition of cyclopean shapes, left visual field (right hemisphere) superiority was observed when the stereogram was comprised of small dots; with large dots, performance was better in the right visual field (left hemisphere). The study also showed that increasing the dot size in the random background could slightly, but significantly, enhance distinguishment accuracy with smaller binocular disparity [11]. Henriksen adopted a stereogram named half-matched to detect the stereo threshold. The research showed that large dots produced stronger responses than small dots, and psychophysical performance decreased with smaller dot size [10].

For the most part, the smallest dot size in RDSs of previous literature was larger than normal recognizability (Snellen VA) of 1 min arc, such as 2.2 min arc in Gray’s study [15], 1.8 × 2.7 in min arc in Ito’s study [16], 3.5 min arc in Tortter’s research [17], and 5 min arc in Stevenson’ work [18], etc.. In our experiment, the smallest dot, 1 × 1 px, with the visual angle of 0.17 min arc, was far smaller than recognition resolution of a normal subject. The test result of RDSs composed of extremely small dots was significantly lower than those composed with larger dots. Blur may be one of the reasons. Recognizability is a measurement of the resolution power of the eye to distinguish adjacent points [19]. Assume that two 1 × 1 px black dots are separated by a 1 × 1 px white dot, if an observer can distinguish between these two black dots, his/her VA should not be lower than 0.17 min arc. However, this resolution could not actually be achieved by a normal eye. A subject with normal VA (about 1 min arc) cannot make out these two dots, but may see only one blurred dot. Under this circumstance, the background composed of extremely small random dots may be expressed as a mottled grey background. The blurred retinal image reduces local contrast and the decreased contrast causes a decrement in stereoacuity [20–22]. Schmidt [23] induced blur with the aid of lenses and found stereoacuity deteriorated 1.341 times faster than Snellen acuity tested under binocular blur conditions and 3.77 times faster under the monocular blur. Crowding may be another factor to influence the test result. The minimum separation from adjacent elements, 1 min arc or more could avoid crowding [24]. Obviously, 0.17 min arc (1 px interval) is too small.

The stereopsis measurements carried out in the clinic are not as complicated as those utilized in a laboratory environment. The test patterns are relatively simple and the target symbols are easy to distinguish. After all, young children should not be excluded from the tests. The RDSs used in the clinic vary by dot size, density and shape. Piano et al. [25] studied 5 commonly used RDSs in the clinic and found a significant difference existing between TNO and Frisby stereotests. It is unwise to attempt to use stereotests interchangeably to test subjects.

In our test, eight RDSs were all carried out in the clinic (Table 1). The dot size at 40 cm was greater than or equal to 1 min arc in 6 out of 8 RDSs, while the dot size was close to 1 min arc (0.8 min arc) in the other 2 RDSs. The test result may be affected by reducing the dot size while increasing the test distance. An RDS composed of extremely small dots may underestimate the stereo threshold according to our experiment. However, there was no significant difference in stereopsis when evaluating stereoacuity with two different dot sizes (6-px versus 10-px). This consequence was different from the literature mentioned above. The discrepancy may result from the different test pattern designs, different test procedures, and different test environments. This reminds us, whether designing or using RDS to test stereopsis, the smallest dot size in the background should be larger than standard subject recognizability at test distance. Otherwise, underestimation of stereo threshold may result. Nevertheless, larger dot size is not necessarily better. Monocular clues become obvious with larger dots because the outline of the target symbol tends to be discerned in this circumstance.

The limitation of our study is that the participants recruited were in a relatively narrow age range, which may lead to possible age bias. All of them were young doctors, nurses and students from our department. Another limitation was that the relative influence factors, such as dot density, were not included in the study. More thorough research should be conducted in future.

## Conclusion

Although disparities in stereotarget settings of a RDS are the test unit used to evaluate the stereothreshold, the effect of background dot sizes should not be neglected. It is recommended to avoid adopting random dot sizes that are smaller than the recognizability of the subject. Otherwise, doing so would result in the underestimation of stereothreshold.

## Supplementary information

**Additional file 1:.**

## Data Availability

All the raw data of this article is shown in additional file 1: Raw data (stereoacuity). xls. The data of personal identity information will not be made available in order to protect the participants’ privacy.

## Abbreviations

arcsec: Second of arc
BCVA: Best corrected visual acuity
MAR: Minimum angle of resolution
min: Minute
px: Pixel
RDS: Random-dot stereograms
TNO: The Netherlands Optical Society
